# L-2-Oxothiazolidine-4-Carboxylic Acid or α-Lipoic Acid Attenuates Airway Remodeling: Involvement of Nuclear Factor-κB (NF-κB), Nuclear Factor Erythroid 2p45-Related Factor-2 (Nrf2), and Hypoxia-Inducible Factor (HIF)

**DOI:** 10.3390/ijms13077915

**Published:** 2012-06-25

**Authors:** Seoung Ju Park, Kyung Sun Lee, Su Jeong Lee, So Ri Kim, Seung Yong Park, Myoung Shin Jeon, Heung Bum Lee, Yong Chul Lee

**Affiliations:** Department of Internal Medicine, Research Center for Pulmonary Disorders, Chonbuk National University Medical School, Jeonju 561-180, South Korea; E-Mails: sjp@jbnu.ac.kr (S.J.P.); kslee3354@hanmail.net (K.S.L.); 2-su@hanmail.net (S.J.L.); sori@jbnu.ac.kr (S.R.K.); cough@jbnu.ac.kr (S.Y.P.); runa0826@nate.com (M.S.J.); lhbmd@jbnu.ac.kr (H.B.L.)

**Keywords:** airway remodeling, antioxidant, hypoxia-inducible factor, nuclear factor erythroid 2p45-related factor-2, nuclear factor-κB, oxidative stress

## Abstract

Reactive oxygen species (ROS) play a crucial role in the pathogenesis of acute and chronic respiratory diseases. Antioxidants have been found to ameliorate airway inflammation and hyperresponsiveness in animal models employing short-term exposure to allergen. However, little data are available on the effect of antioxidants on airway remodeling and signaling pathways in chronic asthma. In the present study, we used a long-term exposure murine model of allergic airway disease to evaluate the effects of an antioxidant, L-2-oxothiazolidine-4-carboxylic acid (OTC) or α-lipoic acid (LA) on airway remodeling, focusing on the ROS-related hypoxia-inducible signaling. Long-term challenge of ovalbumin (OVA) increased ROS production, airway inflammation, and airway hyperresponsiveness, and developed features of airway remodeling such as excessive mucus secretion, subepithelial fibrosis, and thickening of the peribronchial smooth muscle layer. Administration of OTC or LA reduced these features of asthma, including airway remodeling, which was accompanied by suppression of transforming growth factor-β1, vascular endothelial growth factor, and T-helper 2 cytokines. In addition, OVA-induced activation of nuclear factor-κB (NF-κB), nuclear factor erythroid 2p45-related factor-2 (Nrf2), hypoxia-inducible factor (HIF)-1α, and HIF-2α was reduced by OTC or LA. Our results also showed that OTC or LA down-regulated phosphoinositide 3-kinase activity and decreased phosphorylation of p38 mitogen-activated protein kinase but not extracellular signal-regulated kinase 1/2 or c-Jun *N*-terminal kinase. These findings demonstrate that OTC and LA can inhibit activation of NF-κB, Nrf2, and HIF, leading to attenuate allergen-induced airway remodeling.

## 1. Introduction

Asthma is a chronic inflammatory lung disorder characterized by airway hyperresponsiveness (AHR), airflow limitation, and airway remodeling that consists of goblet cell hyperplasia, subepithelial fibrosis, smooth muscle hypertrophy and hyperplasia, and neovascularization [1]. Although reversibility of airway obstruction has traditionally formed part of the definition of asthma, it is apparent that airway obstruction in asthma is often irreversible associating with development of structural changes in the airway. This airway remodeling is speculated to be one of the factors that make difficult to treat asthmatic patients and therefore may be a target for future therapies.

An imbalance between reactive oxygen species (ROS) and the antioxidant defense system leads to oxidative stress, which is closely linked to the pathogenesis of acute and chronic airway disorders [2]. Increased levels of ROS cause direct tissue injury and promote inflammatory responses through regulation of diverse pro-inflammatory mediators in the lung. Furthermore, oxidative stress leads to an increase in airway microvascular permeability and excessive mucus secretion and may alter remodeling of extracellular matrix and blood vessels [3].

The response of a cell to excessive ROS involves activation of multiple signaling pathways, which can cause transcriptional changes and consequently exhibit a variety of activities [4]. Nuclear factor-κB (NF-κB) is one of the major transcription factors that are activated in the lung during oxidative stress, leading to up-regulation of numerous pro-inflammatory genes such as T-helper 2 (Th2) cytokines influencing airway remodeling [5–7]. ROS also increase the transcriptional activity of nuclear factor E2-related factor 2 (Nrf2) and hypoxia-inducible factor (HIF)-1α, resulting in greater expression of their target genes [8–10]. Moreover, recent studies have demonstrated that HIF-1α signaling plays an important role in airway remodeling processes including goblet cell hyperplasia [11,12].

Antioxidant enzymes and proteins are crucial for maintaining the reducing environment of the cell and preventing the oxidative damage. Because glutathione (GSH) synthesized from cysteine is a vital protective antioxidant against oxidative stress, alterations in alveolar and lung GSH metabolism are widely recognized as a central feature of asthma and other airway inflammatory diseases [2]. A thiazolidine derivative, L-2-oxothiazolidine-4-carboxylic acid (OTC) is a prodrug of cysteine that raises the concentrations of cysteine and GSH [13]. α-Lipoic acid (LA) is reduced intracellularly to dihydrolipoic acid by lipoamide dehydrogenase. Dihydrolipoic acid is a highly reactive thiol capable of reducing glutathione disulfide (GSSG) to GSH as well as affecting the oxidation state of thioredoxin and other thiol-containing proteins [14]. We have previously shown that OTC and LA reduce airway inflammation and AHR in a murine model employing short-term exposure to aerosolized allergen [5,15]. However, there are little data on the influence and molecular mechanisms of these antioxidants on chronic allergen-induced airway remodeling, specifically in those associated with the regulation of transcriptional activities.

In the present study, we evaluated the ability of antioxidants, OTC and LA to alter airway remodeling and further explored underlying signaling pathways of their anti-remodeling effect using a long-term exposure murine model of allergic airway disease. We found that OTC and LA successfully improved the airway remodeling and inhibited the ROS-related activation of NF-κB, Nrf2, and HIF as well as the phosphorylation of phosphoinositide 3-kinase (PI3K)/Akt and p38 mitogen-activated protein kinase (MAPK) in mice.

## 2. Results

### 2.1. OTC or LA Decreases ROS Generation and Increases GSH Levels in the Lung

**Figure 1:**
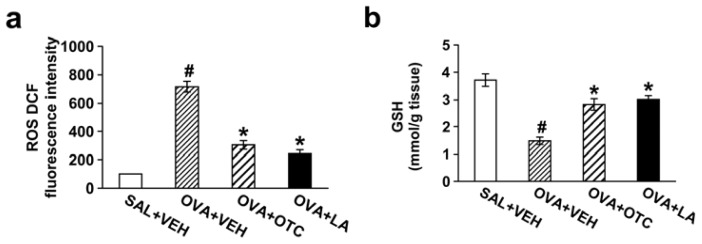

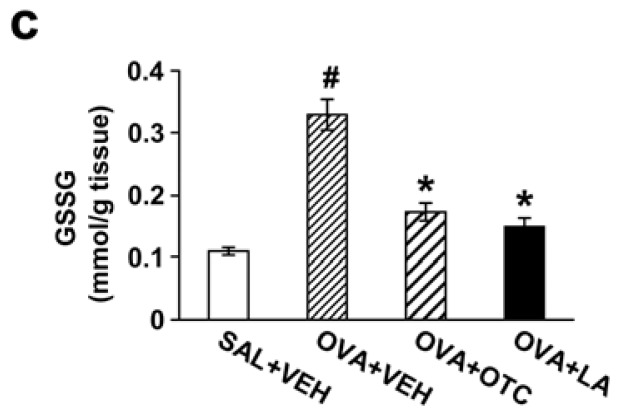
Effect of L-2-oxothiazolidine-4-carboxylic acid (OTC) or α-lipoic acid (LA) on reactive oxygen species (ROS) levels in bronchoalveolar lavage (BAL) cells and glutathione (GSH) and glutathione disulfide (GSSG) levels in lung tissues. Sampling was performed at 48 h after the last challenge in saline-inhaled mice administered drug vehicle (PBS) (SAL + VEH), ovalbumin (OVA)-inhaled mice administered drug vehicle (OVA + VEH), OVA-inhaled mice administered OTC (OVA + OTC), and OVA-inhaled mice administered LA (OVA + LA). (**a**) 2′,7′-dichlorofluorescein (DCF) fluorescence intensity is presented as the relative ratio of ROS levels in OVA + VEH, OVA + OTC, or OVA + LA to those in SAL + VEH. The relative ratio of ROS levels in the BAL fluids of SAL + VEH is arbitrarily presented as 100; (**b**) GSH levels in lung tissues were determined at 48 h after the last challenge; (**c**) GSSG levels in lung tissues were determined at 48 h after the last challenge. Data represent mean ± SEM from 9 mice per group. ^#^ *p* < 0.05 *versus* SAL + VEH; * *p* < 0.05 *versus* OVA + VEH.

To verify the ability of OTC or LA to regulate ROS generation and GSH system in our current murine model of chronic allergic airway disease, we determined ROS levels in bronchoalveolar lavage (BAL) cells and GSH or GSSG levels in lung tissues. ROS levels in BAL cells were significantly increased at 48 h after the last ovalbumin (OVA) inhalation compared with the levels in the control mice (Figure 1a). The increase in ROS generation was substantially reduced by administration of OTC or LA.

Chronic OVA-challenged mice displayed significantly lower GSH levels in lung tissues compared with the levels in the control mice (Figure 1b). The decrease in GSH levels was inhibited by administration of OTC or LA. In contrast, GSSG levels in lung tissues were increased after OVA inhalation and markedly decreased by treatment with OTC or LA (Figure 1c).

### 2.2. OTC and LA Inhibit Allergen-Induced Airway Remodeling

**Figure 2:**
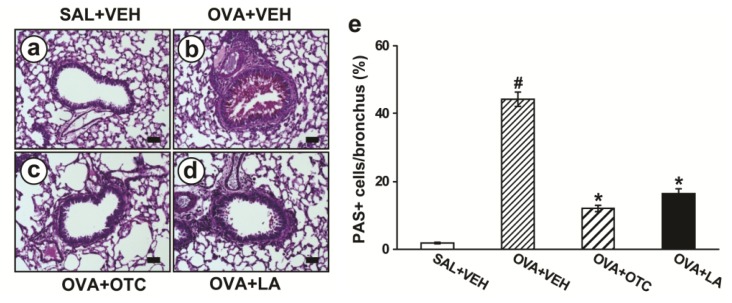
Effect of OTC or LA on airway mucus expression. Sampling was performed at 48 h after the last challenge in saline-inhaled mice administered drug vehicle (SAL + VEH), OVA-inhaled mice administered drug vehicle (OVA + VEH), OVA-inhaled mice administered OTC (OVA + OTC), and OVA-inhaled mice administered LA (OVA + LA). (**a**–**d**) Representative periodic acid-Schiff (PAS)-stained sections of the lungs from SAL + VEH (**a**); OVA + VEH (**b**); OVA + OTC (**c**); and OVA + LA (**d**); The red color indicates PAS-positive mucus expression. Bars indicate scale of 50 μm; (**e**) Quantitation of airway mucus expression. Data represent mean ± SEM from 9 mice per group. ^#^ *p* < 0.05 *versus* SAL + VEH; * *p* < 0.05 *versus* OVA + VEH.

As main characteristics of airway remodeling are mucus hypersecretion, subepithelial fibrosis, and increased smooth muscle mass around airways, we evaluated the effect of OTC and LA on these parameters in allergen-challenged animals. Percentage of airway epithelium which stained positively with periodic acid-Schiff (PAS) was significantly greater in mice repetitively challenged with OVA (Figure 2b) than in the control mice (Figure 2a). Administration of OTC (Figure 2c) or LA (Figure 2d) to OVA-challenged mice dramatically dampened the percentage of airway epithelium staining positively with PAS compared with untreated mice.

**Figure 3:**
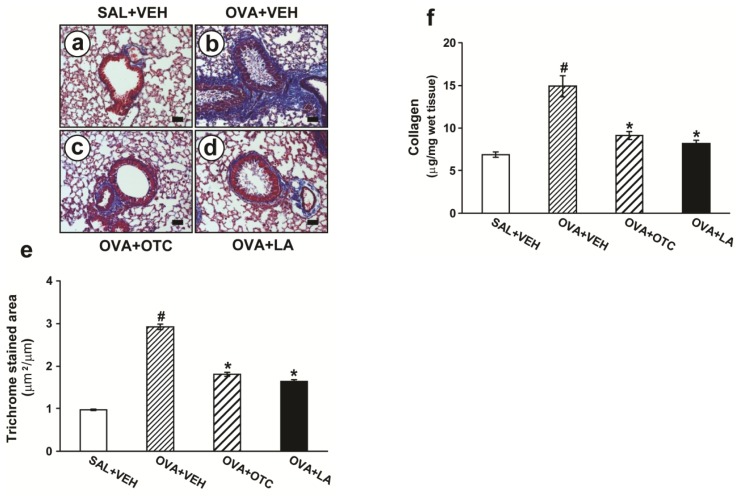
Effects of OTC or LA on peribronchial fibrosis and total lung collagen content. Sampling was performed at 48 h after the last challenge in saline-inhaled mice administered drug vehicle (SAL + VEH), OVA-inhaled mice administered drug vehicle (OVA + VEH), OVA-inhaled mice administered OTC (OVA + OTC), and OVA-inhaled mice administered LA (OVA + LA). (**a**–**d**) Representative peribronchial and perivascular trichrome-stained sections of the lungs from SAL + VEH (**a**); OVA + VEH (**b**); OVA + OTC (**c**); and OVA + LA (**d**). The blue color indicates peribronchial trichrome-stained collagen deposition/fibrosis. Bars indicate scale of 50 μm; (**e**) Quantitation of peribronchial fibrosis. Results are expressed as the area of trichrome staining per micrometer length of basement membrane of bronchioles; (**f**) Total lung collagen content. The amount of lung collagen was measured using a collagen assay kit. Data represent mean ± SEM from 9 mice per group. ^#^ *p* < 0.05 *versus* SAL + VEH; * *p* < 0.05 *versus* OVA + VEH.

Long-term OVA challenge of mice markedly increased levels of peribronchial fibrosis compared with the control mice as assessed by trichrome staining (Figure 3a–e) and determination of total lung collagen content (Figure 3f). When the OVA-inhaled mice were treated with OTC or LA, the levels of peribronchial fibrosis were remarkably reduced.

**Figure 4:**
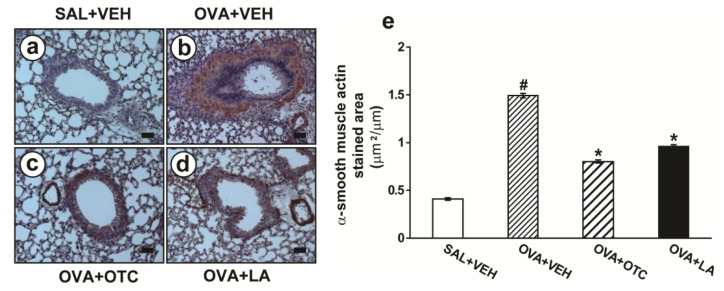
Effects of OTC or LA on airway smooth muscle hyperplasia. Sampling was performed at 48 h after the last challenge in saline-inhaled mice administered drug vehicle (SAL + VEH), OVA-inhaled mice administered drug vehicle (OVA + VEH), OVA-inhaled mice administered OTC (OVA + OTC), and OVA-inhaled mice administered LA (OVA + LA). (**a**–**d**) Representative immunohistochemical-stained sections for α-smooth muscle actin of the lungs from SAL + VEH (**a**); OVA + VEH (**b**); OVA + OTC (**c**); and OVA + LA (**d**); Bars indicate scale of 50 μm. (**e**) The area of peribornchial α-smooth muscle actin immunostaining. Results are expressed as the area of α-smooth muscle actin immunostaining per micrometer length of basement membrane of bronchioles. Data represent mean ± SEM from 9 mice per group. ^#^ *p* < 0.05 *versus* SAL + VEH; * *p* < 0.05 *versus* OVA + VEH.

Mice exposed to repetitive OVA challenge had a significant increase in the area of peribronchial α-smooth muscle actin immunostaing (Figure 4b) compared with the control mice (Figure 4a). Administration of OTC (Figure 4c) or LA (Figure 4d) substantially decreased the thickness of peribronchial smooth muscle layer.

### 2.3. OTC and LA Suppress TGF-β1 Expression in the Lung

**Figure 5:**
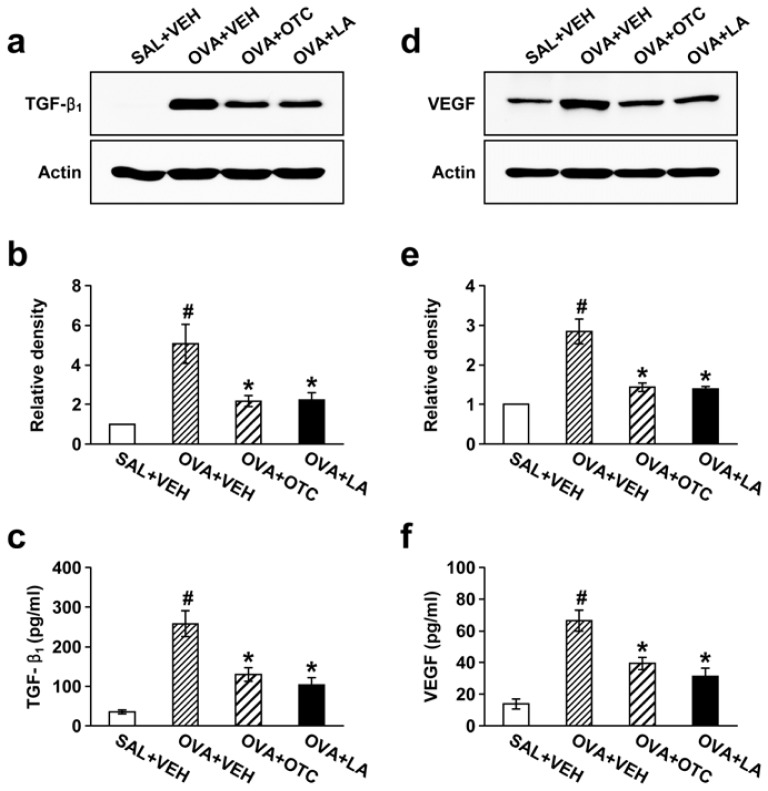
Effect of OTC or LA on the expression of TGF-β1 and vascular endothelial growth factor (VEGF) in lung tissues and in BAL fluids. Sampling was performed at 48 h after the last challenge in saline-inhaled mice administered drug vehicle (SAL + VEH), OVA-inhaled mice administered drug vehicle (OVA + VEH), OVA-inhaled mice administered OTC (OVA + OTC), and OVA-inhaled mice administered LA (OVA + LA). (**a**) Representative Western blotting of TGF-β1; (**b**) Densitometric analyses are presented as the relative ratio of TGF-β1 to actin. The relative ratio of TGF-β1 in the lung tissues of SAL + VEH is arbitrarily presented as 1; (**c**) Enzyme immunoassay of TGF-β1 in BAL fluids; (**d**) Representative Western blotting of VEGF in lung tissues; (**e**) Densitometric analyses are presented as the relative ratio of VEGF to actin. The relative ratio of VEGF in the lung tissues of SAL + VEH is arbitrarily presented as 1; (**f**) Enzyme immunoassay of VEGF in BAL fluids. Data represent mean ± SEM from 9 mice per group. ^#^ *p* < 0.05 *versus* SAL + VEH; * *p* < 0.05 *versus* OVA + VEH.

Since TGF-β1 is a key mediator involved in tissue remodeling in asthma [16], we ascertained whether OTC or LA could attenuate TGF-β1 expression in lung tissues and BAL fluids from chronic OVA-challenged mice. Western blot analysis showed that TGF-β1 protein levels in lung tissues were markedly increased at 48 h after the last OVA inhalation compared with the levels in the control mice (Figure 5a,b). Treatment of mice with OTC or LA resulted in a significant reduction in TGF-β1 levels in lung tissues. Consistent with the results obtained from the Western blot analysis, enzyme immunoassays revealed that the OVA-induced increase in TGF-β1 levels in BAL fluids was strongly inhibited by administration of OTC or LA (Figure 5c).

### 2.4. OTC and LA Inhibit OVA-Induced Vascular Endothelial Growth Factor (VEGF) Expression in the Lung

Given the fundamental role of VEGF in structural changes of the airway wall [17], we assessed whether antioxidants modulate VEGF expression in the lung of our allergen-exposed murine model. Western blot analysis showed that OVA-challenged mice displayed significantly higher VEGF protein levels in lung tissues than the levels in the control mice and that the increase in VEGF levels was substantially repressed by administration of OTC or LA (Figure 5d,e). Consistently, enzyme immunoassay also showed that treatment with OTC or LA remarkably reduced OVA-induced increase in VEGF levels in BAL fluids (Figure 5f).

### 2.5. OTC and LA Suppress Expression of Th2 Cytokines

**Figure 6:**
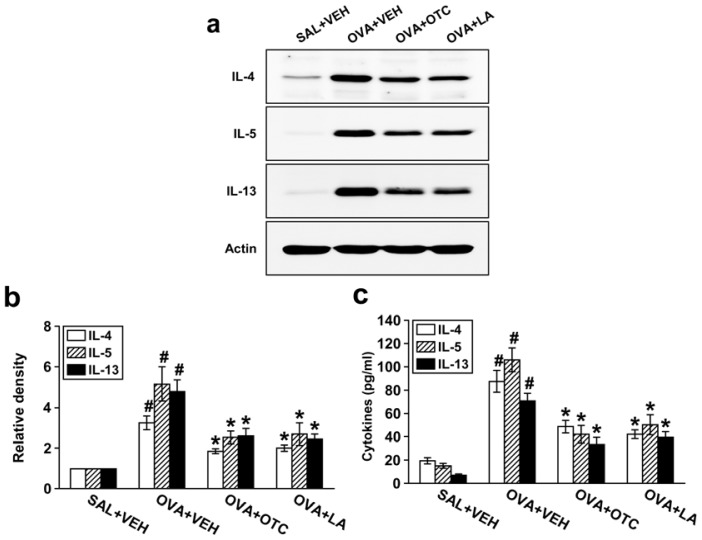
Effect of OTC or LA on IL-4, IL-5, and IL-13 protein levels in lung tissues and in BAL fluids. Sampling was performed at 48 h after the last challenge in saline-inhaled mice administered drug vehicle (SAL + VEH), OVA-inhaled mice administered drug vehicle (OVA + VEH), OVA-inhaled mice administered OTC (OVA + OTC), and OVA-inhaled mice administered LA (OVA + LA). (**a**) The expression of IL-4, IL-5, and IL-13 in lung tissues was identified by Western blotting; (**b**) Densitometric analyses are presented as the relative ratio of IL-4, IL-5, and IL-13 to actin. The relative ratio of each molecule in the lung tissues of SAL + VEH is arbitrarily presented as 1; (**c**) The levels of IL-4, IL-5, and IL-13 were measured in BAL fluids by enzyme immunoassay. Data represent mean ± SEM from 9 mice per group. ^#^ *p* < 0.05 *versus* SAL + VEH; * *p* < 0.05 *versus* OVA + VEH.

Allergic airway inflammation is largely a Th2-driven phenomenon and Th2 cytokines may contribute to structural changes of airway remodeling [18]. We thus tested the effect of OTC or LA on Th2 cytokine expression. Western blot analysis revealed that protein levels of IL-4, IL-5, and IL-13 in lung tissues were substantially higher in OVA-inhaled mice than in the control mice (Figure 6a,b). We observed significant decreases in these cytokine levels in lung tissues by administration of OTC or LA. In agreement with these observations, enzyme immunoassay also showed that levels of IL-4, IL-5, and IL-13 in BAL fluids were profoundly increased after OVA inhalation and that OTC or LA dramatically reduced these increases in Th2 cytokine levels of BAL fluids (Figure 6c).

### 2.6. OTC and LA Negatively Modulate OVA-Induced Activation of NF-κB p65 and Nrf2

**Figure 7:**
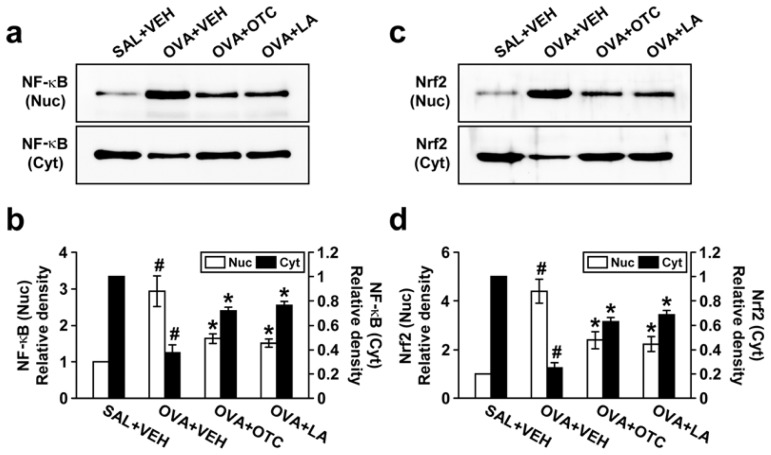
Effect of OTC or LA on levels of NF-κB p65 and Nrf2 in lung tissues. Sampling was performed at 48 h after the last challenge in saline-inhaled mice administered drug vehicle (SAL + VEH), OVA-inhaled mice administered drug vehicle (OVA + VEH), OVA-inhaled mice administered OTC (OVA + OTC), and OVA-inhaled mice administered LA (OVA + LA). (**a**) Representative Western blot analyses of NF-κB p65 levels in nuclear (Nuc) and cytosolic (Cyt) protein extracts from lung tissues; (**b**) Densitometric analyses are presented as the relative ratio of NF-κB p65 levels in OVA + VEH, OVA + OTC, or OVA + LA to those in SAL + VEH; (**c**) Western blot analyses of Nrf2 levels in nuclear and cytosolic protein extracts from lung tissues; (**d**) Densitometric analyses are presented as the relative ratio of Nrf2 levels in OVA + VEH, OVA + OTC, or OVA + LA to those in SAL + VEH. The relative ratio of NF-κB (**b**) or Nrf2 (**d**) in nuclear protein extracts from the lung tissues of SAL + VEH is arbitrarily presented as 1. Data represent mean ± SEM from 9 mice per group. ^#^ *p* < 0.05 *versus* SAL + VEH; * *p* < 0.05 *versus* OVA + VEH.

We next sought to determine whether antioxidants influence the nuclear translocation of NF-κB or Nrf2 in chronic OVA-challenged mice. Levels of both NF-κB p65 and Nrf2 in nuclear protein extracts from lung tissues were dramatically increased after OVA inhalation compared with the levels in the control mice (Figure 7). The increases in these transcription factors in nuclear protein extracts were remarkably decreased by administration of OTC or LA. Conversely, chronic OVA challenge led to substantial decreases in NF-κB p65 and Nrf2 levels in cytosolic protein fractions from lung tissues. The levels of NF-κB p65 and Nrf2 in cytosolic protein fractions were greatly enhanced by treatment with OTC or LA.

### 2.7. OTC and LA Decrease Levels of HIF-1α and HIF-2α

**Figure 8:**
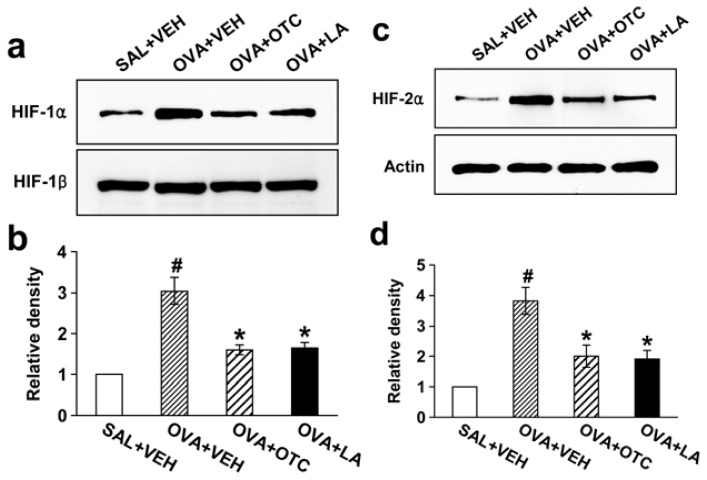
Effect of OTC or LA on levels of hypoxia-inducible factor (HIF)-1α, HIF-1β, and HIF-2α in nuclear protein extracts from lung tissues. Sampling was performed at 48 h after the last challenge in saline-inhaled mice administered drug vehicle (SAL + VEH), OVA-inhaled mice administered drug vehicle (OVA + VEH), OVA-inhaled mice administered OTC (OVA + OTC), and OVA-inhaled mice administered LA (OVA + LA). (**a**) Representative Western blotting of HIF-1α and HIF-1β; (**b**) Densitometric analyses are presented as the relative ratio of HIF-1α to HIF-1β. The relative ratio of HIF-1α in the lung tissues of SAL + VEH is arbitrarily presented as 1; (**c**) Representative Western blotting of HIF-2α; (**d**) Densitometric analyses are presented as the relative ratio of HIF-2α to actin. The relative ratio of HIF-2α in the lung tissues of SAL + VEH is arbitrarily presented as 1. Data represent mean ± SEM from 9 mice per group. ^#^ *p* < 0.05 *versus* SAL + VEH; * *p* < 0.05 *versus* OVA + VEH.

It is well documented that HIF pathway activates transcription of the gene encoding VEGF and is involved in the pathogenesis of asthma [19]. To elucidate whether allergen-induced activation of HIF-1α and HIF-2α is altered by treatment of OTC or LA, we measured HIF-1α and HIF-2α protein levels in nuclear protein extracts from lung tissues using Western blotting. Both isoforms of HIF-α subunit were greatly increased after chronic OVA inhalation compared with the levels in the control mice (Figure 8). The increases in HIF-1α and HIF-2α levels in nuclear protein extracts were substantially decreased by administration of OTC or LA.

### 2.8. OTC and LA Down-Regulate OVA-Induced PI3K/Akt

**Figure 9:**
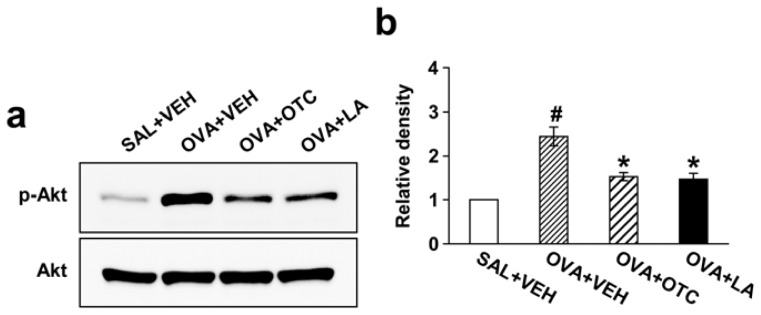
Effect of OTC or LA on p-Akt and Akt protein expression in lung tissues. (**a**) Western blotting of p-Akt and Akt in lung tissues. p-Akt and Akt protein expression was measured at 48 h after the last challenge in saline-inhaled mice administered drug vehicle (SAL + VEH), OVA-inhaled mice administered drug vehicle (OVA + VEH), OVA-inhaled mice administered OTC (OVA + OTC), and OVA-inhaled mice administered LA (OVA + LA); (**b**) Densitometric analyses are presented as the relative ratio of p-Akt to Akt. The relative ratio of p-Akt in the lung tissues of SAL + VEH is arbitrarily presented as 1. Data represent mean ± SEM from 9 mice per group. ^#^ *p* < 0.05 *versus* SAL + VEH; * *p* < 0.05 *versus* OVA + VEH.

We then evaluated which protein kinase mediates the inhibitory effect of antioxidants on airway remodeling in our chronic model of allergic airway disease. In order to address the inhibitory effect of OTC or LA on PI3K/Akt signaling, we determined phosphorylation of Akt using Western blotting. Levels of phosphorylated Akt (p-Akt) protein in lung tissues were significantly increased after repetitive OVA challenge compared with levels in the control mice (Figure 9). However, no significant changes in Akt protein levels were observed in any of the groups tested. The increased p-Akt were significantly reduced by administration of OTC or LA but Akt protein levels were not.

### 2.9. OTC and LA Suppress Phosphorylation of p38 MAPK but Not Extracellular Signal-Regulated Kinase 1/2 (ERK1/2) and c-Jun N-Terminal Kinase (JNK)

**Figure 10:**
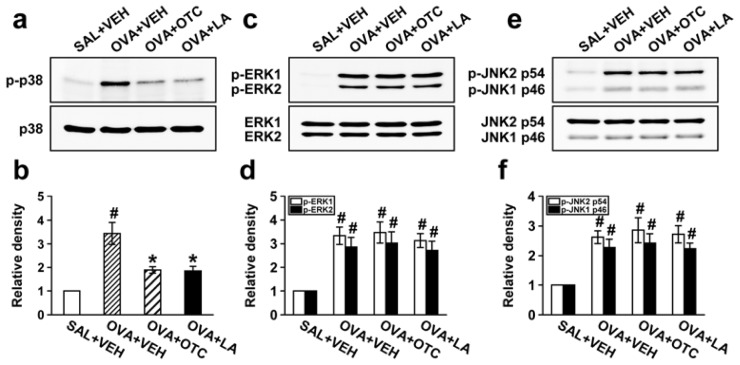
Effect of OTC or LA on phosphorylation of p38 mitogen-activated protein kinase (MAPK), extracellular signal-regulated kinase 1/2 (ERK1/2), and c-Jun *N*-terminal kinase (JNK) in lung tissues. Sampling was performed at 48 h after the last challenge in saline-inhaled mice administered drug vehicle (SAL + VEH), OVA-inhaled mice administered drug vehicle (OVA + VEH), OVA-inhaled mice administered OTC (OVA + OTC), and OVA-inhaled mice administered LA (OVA + LA). (**a**) Representative Western blotting of p-p38 MAPK and p38 MAPK in lung tissues; (**b**) Densitometric analyses are presented as the relative ratio of p-p38 MAPK to p38 MAPK; (**c**) Representative Western blotting of p-ERK1/2 and ERK1/2 in lung tissues; (**d**) Densitometric analyses are presented as the relative ratio of p-ERK1/2 to ERK1/2; (**e**) Representative Western blotting of p-JNK and JNK in lung tissues; (**f**) Densitometric analyses are presented as the relative ratio of p-JNK to JNK. The relative ratio of p-p38 MAPK (**b**), p-ERK1/2 (**d**), and JNK (**f**) in the lung tissues of SAL + VEH is arbitrarily presented as 1. Data represent mean ± SEM from 9 mice per group. ^#^ *p* < 0.05 *versus* SAL + VEH; * *p* < 0.05 *versus* OVA + VEH.

The role of MAPKs in asthma is emerging, and inhibition of MAPK signaling pathway has been found to control the processes of tissue remodeling related to airway inflammation [20]. To assess an involvement of MAPK family in the attenuating effect of antioxidants on airway remodeling, we examined the levels of p38 MAPK, ERK1/2, and JNK and their phosphorylated forms in lung tissues of chronic OVA-challenged mice treated with OTC or LA. Prolonged OVA challenge elicited phosphorylation of p38 MAPK, ERK1/2, and JNK (Figure 10). The increase in p38 MAPK phosphorylation was significantly reduced by administration of OTC or LA. However, OTC and LA did not alter OVA-induced phosphorylation of ERK1/2 or JNK.

### 2.10. OTC and LA Ameliorate Airway Inflammation and AHR in Chronic OVA-Challenged Mice

**Figure 11:**
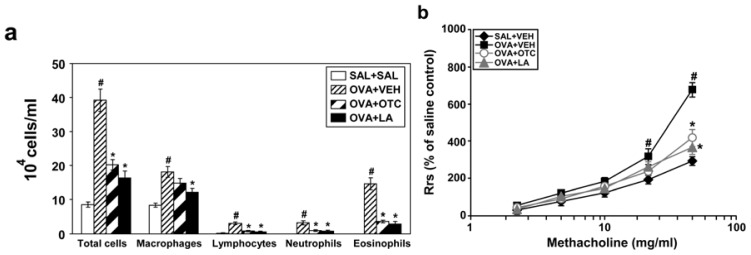
Effect of OTC or LA on total and differential cell counts in BAL fluids and airway responsiveness. (**a**) The numbers of each cellular component of BAL fluids from saline-inhaled mice administered drug vehicle (SAL + VEH), OVA-inhaled mice administered drug vehicle (OVA + VEH), OVA-inhaled mice administered OTC (OVA + OTC), and OVA-inhaled mice administered LA (OVA + LA); (**b**) Airway responsiveness. Data represent mean ± SEM from 9 mice per group. ^#^ *p* < 0.05 *versus* SAL + VEH; * *p* < 0.05 *versus* OVA + VEH.

Since a hallmark of allergic airway disorder is the infiltration of inflammatory cells into the airways, we investigated the effect of antioxidants on numbers of inflammatory cells in BAL fluids. Numbers of total cells, macrophages, lymphocytes, neutrophils, and eosinophils were significantly increased in the BAL fluid at 48 h after the last OVA inhalation compared with the levels in the control mice (Figure 11a). Treatment with OTC or LA markedly decreased the numbers of total cells, lymphocytes, neutrophils, and eosinophils.

To determine the effect of antioxidants on AHR in chronic allergen-challenged mice, airway responsiveness was assessed as a percent increase of respiratory system resistance (Rrs) in response to increasing doses of methacholine. In OVA-sensitized and -challenged mice, the dose-response curve of percent Rrs shifted to the left compared with that of control mice (Figure 11b). In addition, the percent Rrs produced by administration of methacholine (25 mg/mL and 50 mg/mL) was increased significantly in the OVA-challenged mice compared with the controls. OVA-inhaled mice treated with OTC or LA showed a dose-response curve of percent Rrs that shifted to the right and a significant reduction in the percent Rrs produced by methacholine at 50 mg/mL compared with that of untreated mice. These results indicate that OTC or LA treatment alleviates OVA-induced AHR.

## 3. Discussion

While the brilliant contribution of oxidative stress to allergic airway inflammation has been firmly established, the effects of anti-oxidative therapy using pharmacologic agents and the related mechanisms on airway remodeling of asthma are not well understood. Our present study demonstrated that potent antioxidants, OTC and LA effectively inhibit allergen-induced airway remodeling and that the regulation of ROS-mediated transcription factors NF-κB, Nrf2, and HIF can be one of the action mechanisms involved in this effect.

There is accumulating evidence that oxidative stress is implicated in the pathogenesis of asthma [2]. An excessive production of ROS is responsible for tissue injury, airway inflammation, and AHR observed in asthma [21]. As expected, our results showed that ROS generation in BAL cells was significantly increased by repetitive OVA challenges and that OTC and LA remarkably reduced ROS production with attenuating allergic airway inflammation and AHR.

In addition, the lung has several natural antioxidant mechanisms to neutralize overproduced oxidants, which include enzymatic as well as non-enzymatic antioxidants. These antioxidant defense systems form a tightly regulated network to resist any change in the redox environment of intra- and extracellular space [22]. Enzymatic antioxidants include catalase, glutathione peroxidase (GPX) and superoxide dismutase (SOD), and non-enzymatic antioxidants are vitamin C, vitamin E, albumin, uric acid, ceruloplasmin, and GSH [23–25]. Moreover, some antioxidants such as LA have ability to regenerate/recycle endogenous and exogenous antioxidants such as vitamins C and E and GSH. Although there is uncertainty whether OTC or LA has this regenerating/recycling effect on their own anti-oxidative properties, in this study, administration of OTC or LA markedly increased GSH levels in lung tissues of chronic OVA-challenged mice, while reducing GSSG levels. These data are in accordance with previous results in the animal model of acute airway inflammatory disease [5,15], ascertaining that OTC and LA can be effective in ameliorating ROS-mediated airway disease through regulating GSH system in the lung.

Oxidative stress appears to induce structural changes in the airway of asthma [26,27]. Goblet cell hyperplasia is enhanced after epithelial damage by endogenous and exogenous ROS [27,28]. Oxidative stress also amplifies proliferation and hypertrophy of smooth muscle cells in the pulmonary vasculature [29]. Moreover, the imbalance between ROS and antioxidant in the lung can activate TGF-β1, which plays an integral role in the development of subepithelial fibrosis ranging from fibroblast differentiation to deposition of connective tissue [30]. Interestingly, recent studies of allergen-induced airway remodeling using transgenic mice indicate an essential role for Th2 cytokines in asthma-related structural changes [18,31], and ROS are known to stimulate the production of these cytokines [5]. Moreover, our previous study has shown that VEGF, a potent angiogenic factor required for airway remodeling, regulates TGF-β1 expression, which results in subepithelial fibrosis in a murine model of allergic airway disease [32]. In this study, the mice chronically exposed to OVA developed characteristic features of airway remodeling, which was composed of mucus hypersecretion, subepithelial fibrosis, and increased smooth muscle mass around airways. In addition, expression of TGF-β1, VEGF, and Th2 cytokines was dramatically increased after chronic OVA challenge. Altogether, these data are strongly supported by a report demonstrating that a strong antioxidant protein not only prevents the development of the structural changes in airways but also alleviates the established airway remodeling [27].

Emerging evidence indicates that oxidative stress is related to regulation of multiple signaling pathways including transcription factors [4]. In the present study, repetitive allergen inhalation triggered the translocation of both NF-κB and Nrf2 from the cytoplasm to nucleus, which was inhibited by treatment with OTC and LA. Modulation of NF-κB pathway in chronic asthma would be of particular importance, since NF-κB-dependent responses are considered to be pivotal pathophysiological processes of airway remodeling [18]. NF-κB induced the enhanced gene transcription of wide range of cytokines, chemokines, and growth factors such as TGF-β1 and IL-13 that contribute to inflammation and remodeling of the airway. Additionally, Nrf2 has been found to possess a role in the lung inflammation, injury, and fibrosis [9,33]. Based on these observations, we suggest that inhibition of NF-κB and Nrf2 by OTC and LA leads to suppression of several target genes and resultant anti-inflammatory and anti-remodeling properties on allergic airway.

VEGF is one of genes whose expression is regulated by HIF, a heterodimeric transcription factor consisting of α subunit (HIF-1α and HIF-2α) and β subunit [34,35]. Recent reports have demonstrated a potential role of HIF-1 induction in vascular remodeling [36] and ROS as essential intermediates for HIF signaling in non-hypoxic conditions [37]. In keeping with these observations, we have found that VEGF expression was up-regulated and HIF-1α and HIF-2α levels in nuclear extracts were substantially increased in response to chronic allergen inhalation associated with excessive ROS generation. All these increases in levels of VEGF, HIF-1α, and HIF-2α were significantly reduced by administration of OTC or LA. Thus, we suggest that one likely mechanism for effects of OTC or LA on airway remodeling could be the modulation of HIF/VEGF pathway.

Phosphorylation plays a key role in the regulation of transcriptional activity of HIF-1α, NF-κB, and Nrf2 [38–43]. In fact, PI3K up-regulates Akt-mediated NF-κB activation in the lung [38,39] and the transcriptional activation of Nrf2 is regulated by PI3K and MAPK [40]. In addition, a recent study has revealed that HIF-1α activation is mediated in part by PI3K-Akt and NF-κB pathways in bronchial epithelial cells [12]. As expected, our results showed the significant activation of PI3K/Akt and MAPK pathways in an animal model that exhibits many pathogenic features similar to those of chronic asthma. Interestingly, while OTC and LA substantially inhibited the activation of PI3K/Akt and p38MAPK, these agents showed no significant effects on the levels of phosphorylation of ERK1/2 and JNK in lung tissues of mice. Taken together, we suggest that OTC and LA inhibit activation of NF-κB, Nrf2, and HIF-1α which may be mediated by PI3K/Akt and p38 MAPK pathways, thereby decreasing expression of various molecules that are involved in airway remodeling.

Lastly, a very recent study has demonstrated that fibroblast proliferation and collagen expression in the lung induced by a fibrogenic agent are regulated by ROS-mediated PI3K/Akt signaling, suggesting a novel role of PI3K/Akt in structural changes of the lung [44]. Hence, OTC and LA may reduce airway remodeling at least in part via down-regulation of PI3K/Akt pathway. Furthermore, PI3K serves as an upstream regulator of VEGF expression through activation of HIF [45], it is also possible that antioxidants may act via modulation of PI3K→HIF→VEGF pathway in allergic airway disease.

## 4. Experimental Section

### 4.1. Animals and Experimental Protocol

**Figure 12:**
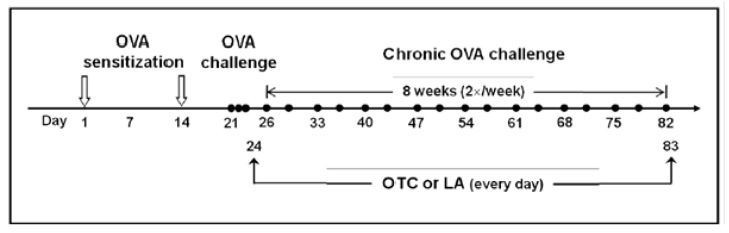
Schematic diagram of the experimental protocol. Mice were sensitized on days 1 and 14 by intraperitoneal injection of 20 μg OVA emulsified in 1 mg of aluminum hydroxide. On days 21, 22, and 23 after the initial sensitization, the mice were challenged for 30 min with an aerosol of 3% (weight/volume) OVA, and then repeated twice a week for 8 weeks beginning on day 26 with an aerosol of 1% (weight/volume) OVA in saline (or with saline as a control) using an ultrasonic nebulizer. In the case of treatment with OTC, it was administered intraperitoneally at 24-h intervals on days 24–83, beginning 4 days after the first challenge. In the case of treatment with LA, it was given by oral gavage at 24-h intervals on days 24–83, beginning 4 days after the first challenge.

Female C57BL/6 mice, 6 weeks of age and free of murine specific pathogens, were obtained from the Orientbio Inc. (Seoungnam, Korea), were housed throughout the experiments in a laminar flow cabinet, and were maintained on standard laboratory chow ad libitum. All experimental animals used in this study were under a protocol approved by the Institutional Animal Care and Use Committee of the Chonbuk National University. Standard guidelines for laboratory animal care were followed [46]. Mice were sensitized on days 1 and 14 by intraperitoneal injection of 20 μg OVA (Sigma-Aldrich, St. Louis, MO) emulsified in 1 mg of aluminum hydroxide (Pierce Chemical Co., Rockford, IL, USA) in a total volume of 200 μL, as previously described with some modifications [47,48] (Figure 12). On days 21, 22, and 23 after the initial sensitization, the mice were challenged for 30 min with an aerosol of 3% OVA (weight/volume), and then repeated twice a week for 8 weeks beginning on day 26 with an aerosol of 1% (weight/volume) OVA in saline (or with saline as a control) using an ultrasonic nebulizer (NE-U12, Omron, Japan). BAL was performed at 48 h after the last challenge. At the time of lavage, the mice (9 mice in each group) were sacrificed by ether inhalation (Junsei Chemical Co., Ltd, Tokyo, Japan). Chest cavity was exposed to allow for expansion, after which the trachea was carefully intubated and the catheter secured with ligatures. Prewarmed 0.9% NaCl solution was slowly infused into the lung and withdrawn. The aliquots were pooled and then kept at 4 °C. A part of each pool was then centrifuged and the supernatants were kept at −70 °C until use. Total cell numbers were counted with a hemocytometer. Smears of BAL cells were prepared with a cytospin (Thermo Electron, Waltham, MA). The smears were stained with Diff-Quik solution (Dade Diagnostics of P. R. Inc. Aguada, Puerto Rico) in order to determine differential cell counts. Two independent, blinded investigators counted the cells using a microscope. Approximately 400 cells were counted in each of four different random locations. Inter-investigator variation was less than 5%. Numbers counted by two investigators were averaged and these values were used to calculate differential cell counts.

### 4.2. Administration of OTC and LA

OTC (160 mg/kg body weight/day, Sigma-Aldrich) was freshly prepared by dissolving the chemical in phosphate buffered saline (PBS) and adjusting pH to 7.2 with 3 N NaOH as described elsewhere [15], and administered intraperitoneally at 24-h intervals on days 24–83, beginning 4 days after the first challenge. LA (100 mg/kg body weight/day, Sigma-Aldrich) dissolved in 1 N NaOH and diluted in PBS as vehicle, which is a nonenzymatic antioxidant, was administered by oral gavage at 24-h intervals on days 24–83, beginning 4 days after the first challenge (Figure 12).

### 4.3. Measurement of Intracellular ROS

ROS were measured by a method previously described [49]. BAL cells were washed with PBS. To measure intracellular ROS, cells were incubated for 10 min at room temperature with PBS containing 3.3 μM 2′,7′-dichlorofluorescein (DCF) diacetate (Molecular probes, Eugene, OR), to label intracellular ROS. We performed fluorescence-activated cell sorting analysis with DCF stained cells (1 × 10^4^ cells) in BAL fluids to measure ROS levels using a FACSCalibur instrument (BD Biosciences, San Jose, CA). The data were analyzed with a CellQuest Pro program (BD Biosciences).

### 4.4. Measurement of GSH and GSSG in Lung Tissues

Lung tissues were homogenized with 10 mL of an ice-cold lysis buffer (50 mM phosphate buffer containing 1 mM ethylene diamine tetraacetic acid (EDTA)) per gram tissue. After centrifugation at 10,000× *g* for 15 min at 4 °C, the supernatants were removed, deproteinated, and then stored at −20 °C until the samples were assayed. Total GSH and GSSG levels were determined using a GSH Assay Kit (Cayman Chemical Company, Ann Arbor, MI) according to the manufacturer’s protocol.

### 4.5. Western Blot Analysis

Lung tissues were homogenized in the lysis buffer containing protease inhibitors and protein concentrations were determined using the Bradford reagent (Bio-Rad Laboratories Inc., Hercules, CA, USA). Samples were loaded on SDS-PAGE gel. After electrophoresis at 120 V for 90 min, separated proteins were transferred to polyvinylidene difluoride membranes (GE Healthcare, Little Chalfont, Buckinghamshire, United Kingdom) by the wet transfer method (250 mA, 90 min). Nonspecific sites were blocked with 5% non-fat dry milk in Tris-buffered saline with Tween 20 (25 mM Tris, pH 7.5, 150 mM NaCl, 0.1% Tween 20) for 1 h, and the blots were then incubated overnight at 4 °C with an anti-TGF-β1 antibody (Sigma-Aldrich), anti-VEGF antibody (Santa Cruz Biotechnology, Santa Cruz, CA), anti-IL-4 antibody (Serotec Ltd., Oxford, United Kingdom), anti-IL-5 antibody (Santa Cruz Biotechnology), anti-IL-13 antibody (R&D Systems, Inc., Minneapolis, MN, USA), anti-Akt antibody (Cell Signaling Technology Inc., Beverly, MA, USA), anti-p-Akt antibody (R&D Systems, Inc.), anti-p38 MAPK antibody (Cell Signaling Technology Inc.), anti-p-p38 MAPK antibody (R&D Systems, Inc.), anti-ERK1/2 antibody (Cell Signaling Technology Inc.), anti-p-ERK1/2 antibody (Cell Signaling Technology Inc.), anti-JNK antibody (Cell Signaling Technology Inc.), or anti-p-JNK antibody (Cell Signaling Technology Inc.). Anti-rabbit or anti-mouse horseradish peroxidase conjugated-IgG was used to detect binding of antibodies. The membranes were stripped and reblotted with an anti-actin antibody (Sigma-Aldrich) to verify equal loading of protein in each lane. The binding of the specific antibodies was visualized by exposing to photographic film after treating with enhanced chemiluminescence system reagents (GE Healthcare).

### 4.6. Cytosolic or Nuclear Protein Extractions for Analysis of NF-κB p65, Nrf2, HIF-1α, HIF-1β, and HIF-2α

Lungs were removed and homogenized in 2 volumes of buffer A (50 mM Tris-HCl, pH 7.5, 1 mM EDTA, 10% glycerol, 0.5 mM dithioltreitol, 5 mM MgCl_2_, and 1 mM phenylmethylsulfonyl fluoride) containing protease inhibitor cocktails. The homogenates were centrifuged at 1000× *g* for 15 min at 4 °C. The supernatant fraction was incubated on ice for 10 min and centrifuged at 100,000× *g* for 1 h at 4 °C to obtain cytosolic proteins for analysis of NF-κB p65. The pellets were washed twice in buffer A and resuspended in buffer B (1.3 mM sucrose, 1.0 mM MgCl_2_, and 10 mM potassium phosphate buffer, pH 6.8) and pelleted at 1000× *g* for 15 min. The pellets were suspended in buffer B with a final sucrose concentration of 2.2 M and centrifuged at 100,000× *g* for 1 h. The resulting pellets were washed once with a solution containing 0.25 M sucrose, 0.5 mM MgCl_2_, and 20 mM Tris-HCl, pH 7.2, and centrifuged at 1000× *g* for 10 min. The pellets were solubilized with a solution containing 50 mM Tris-HCl (pH 7.2), 0.3 M sucrose, 150 mM NaCl, 2 mM EDTA, 20% glycerol, 2% Triton X-100, 2 mM phenylmethylsulfonyl fluoride, and protease inhibitor cocktails. The mixture was kept on ice for 1 h with gentle stirring and centrifuged at 12,000× *g* for 30 min. The resulting supernatants were used as soluble nuclear proteins for analysis of NF-κB p65, Nrf2, HIF-1α, HIF-1β, and HIF-2α. The levels of these proteins were analyzed by Western blotting using antibody against NF-κB p65 (Upstate Biotech, Lake Placid, NY, USA), Nrf2 (Santa Cruz Biotechnology), HIF-1α (R&D Systems, Inc.), HIF-1β (Cell Signaling Technology Inc.), or HIF-2α (Novus Biologicals Inc., Littleton, CO) as described above.

### 4.7. Measurement of TGF-β1, VEGF, and Th2 Cytokines

Levels of TGF-β1, VEGF, IL-4, IL-5, and IL-13 were quantified in the supernatants of BAL fluids by enzyme immunoassays according to the manufacturer’s protocol (TGF-β1; Bender MedSystems, Vienna, Austria, VEGF; R&D Systems, Inc., IL-4 and IL-5; Invitrogen, Carlsbad, CA, and IL-13; Bender MedSystems). Sensitivities for TGF-β1, VEGF, IL-4, IL-5, and IL-13 assays were 9, 3, 5, 3, and 2.8 pg/mL, respectively.

### 4.8. Processing of Lungs for Histologic and Image Analysis

At 48 h after the last challenge, mice were euthanized for histological assessment. The lungs and trachea of mice were filled with 10% (volume/volume) neutral buffered formalin intratracheally and then were removed from the mice. For fixation, the neutral buffered formalin was also used [50]. The specimens were dehydrated and embedded in paraffin. For histological examination, 4-μm sections of fixed embedded tissues were cut on a Leica model 2165 rotary microtome (Leica Microsystems Nussloch GmbH, Nussloch, Germany). The specimens were stained sequentially with PAS, Masson’s trichrome stain, or α-smooth muscle actin stain. Stained and immunostained slides were all quantified under identical light microscope conditions, including magnification (20×), gain, camera position, and background illumination.

### 4.9. Quantitation of Airway Mucus Expression

To quantify the level of mucus expression in the airway, the number of PAS-positive and PAS-negative epithelial cells in individual bronchioles were counted as described previously [50]. Results are expressed as the percentage of PAS-positive cells per bronchiole, which is calculated from the number of PAS-positive epithelial cells per bronchiole divided by the total number of epithelial cells of each bronchiole.

### 4.10. Assessment of Peribronchial Fibrosis

Two methods (trichrome staining and total lung collagen content) were used to quantify peribronchial fibrosis.

#### Peribronchial trichrome staining

The area of peribronchial trichrome staining in a paraffin-embedded lung was outlined and quantified using a light microscope (Leica DM LB; Leica Mikroskopie & Systeme GmbH, Wetzlar, Germany) attached to an image-analysis system (analySIS Pro version 3.2; Soft Imaging System GmbH, Münster, Germany). Results are expressed as the area of trichrome staining per micron length of basement membrane of bronchioles 150–200 μm of internal diameter. At least ten bronchioles were counted in each slide.

#### Determination of total lung collagen content

The total lung collagen content was determined using the Sircol Collagen Assay kit (Biocolor Ltd., Belfast, Northern Ireland) according to the manufacturer’s protocols.

### 4.11. Quantitation of Peribronchial Smooth Muscle Layer Thickness

The area of α-smooth muscle actin staining was outlined and quantified using a light microscope attached to an image-analysis system as described above. Results are expressed as the area of immunostaining per micron length of basement membrane of bronchioles 150–200 μm of internal diameter.

### 4.12. Determination of Airway Responsiveness

Airway responsiveness was assessed as a change in airway function after challenge with aerosolized methacholine via airways, as described elsewhere [49,50]. Anesthesia was achieved with 45 mg/kg of pentobarbital sodium injected intraperitoneally. The trachea was then exposed through midcervical incision, tracheostomized, and an 18-gauge metal needle was inserted. Mice were connected to a computer-controlled small animal ventilator (flexiVent, SCIREQ, Montreal, Canada). The mouse was quasi-sinusoidally ventilated with nominal tidal volume of 10 mL/kg at a frequency of 150 breaths/minute and a positive end-expiratory pressure of 2 cm H_2_O to achieve a mean lung volume close to that during spontaneous breathing. This was achieved by connecting the expiratory port of the ventilator to water column. Methacholine aerosol was generated with an in-line nebulizer and administered directly through the ventilator. To determine the differences in airway response to methacholine, each mouse was challenged with methacholine aerosol in increasing concentrations (2.5–50 mg/mL in saline). After each methacholine challenge, the data of Rrs was continuously collected. Maximum values of Rrs were selected to express changes in airway function, which was represented as a percentage change from baseline after saline aerosol.

### 4.13. Densitometric Analyses and Statistics

All immunoreactive and phosphorylated signals were analyzed by densitometric scanning (Gel Doc XR, Bio-Rad Laboratories Inc.). Data were expressed as mean ± SEM. Statistical comparisons were performed using one-way ANOVA followed by the Scheffe’s test. Statistical significance was set at *p* < 0.05.

## 5. Conclusions

In this study, we have found that oxidative stress including ROS plays a critical role in development of airway remodeling and that the ROS-related processes are required for activation of various transcription factors such as NF-κB, Nrf2, and HIF and protein kinases. Each transcriptional regulation for the target genes contributes to the pathologic components of airway remodeling, that is, inflammatory responses, smooth muscle hyperplasia, and vascular changes cooperatively. In addition, among MAPKs, p38 is only implicated in ROS-induced activation in an airway remodeling model. Together, these findings suggest that ROS-mediated airway remodeling in allergic airway disease is regulated by a network involving various transcriptional regulations and provide evidence that antioxidants can be a very powerful therapeutic agent for chronic airway disorders.
